# Serum gelsolin as a biomarker for differentiating familial Mediterranean fever from juvenile idiopathic arthritis in children

**DOI:** 10.17305/bb.2026.13983

**Published:** 2026-05-04

**Authors:** Esra Dişçi, Amra Adrovic Yıldız, Saime Özbek Şebin, Kamber Kaşali

**Affiliations:** 1 Department of Pediatric Health and Diseases Clinic, Erzurum City Hospital, Erzurum, Türkiye; 2 Department of Pediatric Rheumatology, Marmara University Pendik Training and Research Hospital, Istanbul, Türkiye; 3 Department of Physiology, Faculty of Medicine, Atatürk University, Erzurum, Türkiye; 4 Department of Biostatistics, Faculty of Medicine, Atatürk University, Erzurum, Türkiye

**Keywords:** Gelsolin, bio-marker, pediatric inflammation

## Abstract

Gelsolin (Gsn), an actin-binding protein implicated in cytoskeletal remodeling and the regulation of inflammation, has emerged as a potential biomarker for immune-mediated and inflammatory diseases. This prospective case-control study aimed to evaluate serum Gsn levels in children diagnosed with juvenile idiopathic arthritis (JIA) and familial Mediterranean fever (FMF) and to assess its potential utility in differentiating between these two pediatric rheumatic disorders. The study involved 90 children aged 2–18 years: 30 with JIA, 30 with FMF, and 30 age-matched healthy controls. Serum Gsn concentrations were quantified using enzyme-linked immunosorbent assay (ELISA), and both clinical and laboratory parameters were analyzed. Mean serum Gsn levels were highest in the FMF group (28.8 ± 2.5), followed by the JIA group (25.7 ± 2.8) and healthy controls (23.9 ± 2.7), with significant differences observed among the groups (*P <* 0.001). Gsn levels were significantly elevated in children with FMF compared to those with JIA, and receiver operating characteristic (ROC) analysis demonstrated strong discriminatory performance, with an area under the curve (AUC) of 0.82. Logistic regression analysis identified serum Gsn, aspartate aminotransferase (AST), and lactate dehydrogenase (LDH) as independent predictors for distinguishing FMF from JIA. Each unit increase in Gsn was associated with increased odds of an FMF diagnosis (odds ratio [OR]: 2.89, 95% confidence interval [CI]: 1.58–5.29, *P ═* 0.001). These findings suggest that serum Gsn may serve as a valuable biomarker in pediatric inflammatory rheumatic diseases, particularly in differentiating FMF from JIA. Further large-scale prospective studies are necessary to validate its diagnostic effectiveness.

## Introduction

Juvenile idiopathic arthritis (JIA) is the most prevalent rheumatic disease in childhood, characterized by chronic autoimmune responses that lead to persistent synovial inflammation, progressive cartilage destruction, and bone erosion [1]. The identification of reliable biomarkers for early diagnosis, disease stratification, and prognostic assessment is crucial [2]. While various factors influencing disease progression have been documented, the precise pathophysiological mechanisms linking proposed biomarkers to disease progression remain inadequately understood [2]. Emerging evidence indicates that gelsolin (Gsn) may play a significant role in the pathogenesis of Rheumatoid Arthritis (RA), underscoring its potential relevance in inflammatory joint diseases [2–4].

Familial Mediterranean Fever (FMF) is the most common monogenic autoinflammatory disorder, characterized by exaggerated inflammatory responses mediated by components of the innate immune system. The disease is closely associated with AA amyloidosis, and the identification of mutations in the *MEFV* gene—most frequently located in exon 10—confirms the diagnosis [5, 6]. In hereditary amyloidoses, several proteins contribute to amyloid formation, including transthyretin, apolipoprotein A1, gelsolin, fibrinogen alpha chain, and lysozyme. Structural alterations due to mutations in these proteins facilitate the formation and deposition of amyloid fibrils [6, 7].

FMF is classified as an “autoinflammatory” rheumatic disease, characterized by uncontrolled activation of the innate immune system, whereas JIA is categorized as an “autoimmune” rheumatic disease, wherein the immune system targets body tissues. FMF manifests as recurrent episodes of high fever lasting 1–3 days, accompanied by abdominal, chest, or joint pain, while JIA is marked by persistent joint pain, swelling, and stiffness, particularly in the mornings. FMF is inherited in an autosomal recessive pattern due to mutations in the *MEFV* gene, while the genetic basis of JIA is more complex and multifactorial. Colchicine is commonly prescribed for FMF to prevent attacks and reduce the risk of amyloidosis, whereas JIA is treated with medications aimed at suppressing joint inflammation, such as methotrexate and biological agents. Both conditions are chronic inflammatory disorders affecting children.

Gsn is an 82-kDa actin-binding protein composed of six homologous subdomains (S1–S6) and is activated in a calcium-dependent manner [8]. It serves as a key regulator of actin filament assembly and disassembly, playing a central role in cytoskeletal dynamics [9]. At low intracellular calcium concentrations [Ca^2+^], Gsn exhibits low affinity for actin; however, elevated [Ca^2+^] levels significantly enhance its actin-binding capacity, facilitating actin remodeling during cellular activation and inflammatory responses [8].

Gelsolin is crucial in modulating inflammatory responses through its ability to sever actin filaments, thereby contributing to the localization of inflammation and preventing the systemic dissemination of pro-inflammatory lipids [10]. Additionally, Gsn is actively involved in immune regulation, including macrophage activation and the spatial confinement of inflammatory processes [11]. Variations in Gsn levels have been implicated in the pathogenesis of various diseases, emphasizing its role as a mediator of inflammatory and immune responses [2–4, 6, 7, 12–14].

Despite its biological significance, Gsn levels have been infrequently studied in patients with chronic inflammatory disorders, particularly within the pediatric population [13–17].

FMF is an inherited autoinflammatory disorder characterized by recurrent episodes of fever and serosal inflammation, resulting from mutations in the *MEFV* gene, which disrupt normal inflammatory regulation. JIA, the most common pediatric rheumatologic disease, is characterized by persistent joint inflammation and encompasses multiple subtypes distinguished by clinical and immunologic features [1, 2, 6, 8]. Given its established involvement in inflammatory processes, this study aimed to evaluate the potential utility of Gsn as a biomarker in chronic inflammatory conditions [4, 5].

This study aimed to investigate serum Gsn levels in pediatric patients with JIA and FMF and to explore their potential role as biomarkers of inflammatory disease activity. We hypothesized that serum Gsn levels would be elevated in pediatric inflammatory diseases, with higher levels observed in autoinflammatory conditions such as FMF compared to autoimmune disorders like JIA.

To the best of our knowledge, this study is the first to evaluate serum Gsn levels in children diagnosed with JIA and FMF.

**Table 1:** Association between gelsolin levels and laboratory and demographic parameters in the JIA, FMF, and control groups

		**JIA**		**FMF**		**Control**					
	**Mean±SD (95% CI)**	**Median (min–max)**	**Mean±SD (95% CI)**	**Median (min–max)**	**Mean±SD (95% CI)**	**Median (min–max)**	***P***	**FDR-adjusted *p* value**	**post-hoc**	**Covariate analysis (Age)**	**Eta^2^ (95% CI)**
Age (years)	13 ±4 (10.6–13.7)	13 (4–18)	9 ± 3 (7.8–10.3)	9 (4–15)	9 ± 3 (7.3–9.9)	9 (3–18)	**0.001** ^&^	0.013	Control-JIA, FMF-JIA		0.163 (0.037-0.290)
Weight SDS	−0.2 ± 3.7 (−4.8–1.7)	−0.2 (−48–4.4)	−0.3 ± 1.01 (−0.7–0.02)	−0.3 (−2.6–1.2)	−0.6 ± 0.7 (−0.7– −0.1)	−0.6 (−1.6–1.3)	0.659^&^	0.986		0.258	0.011 (0.000–0.071)
Height SDS	0.1 ± 1.3 (−0.5–0.4)	0.1 (−1.9–4.2)	−0.1 ± 0.9 (−0.7– −0.04)	−0.1 (−2.0–1.8)	−0.08 ± 1 (−0.2–0.5)	−0.08 (−1.4–2.9)	0.174^&^	0.565		0.057	0.042 (0.000–0.135)
BMİ SDS	−0.2 ± 1.4 (−0.6–0.4)	−0.2 (−3–3.5)	−0.1 ± 1.2 (−0.6–0.3)	−0.1 (−2.8–2.1)	−0.8 ± 0.8 (−1.1– −0.4)	−0.8 (−2.2–1.0)	0.076^$^	0.247		0.136	0.057 (0.000–0.159)
BUN (mg/dL)	12 ± 3 (10.4–12.3)	12 (7–19)	11 ± 3 (10.4–12.6)	11 (7–21)	11 ± 3 (10.8–12.9)	11 (8–17)	0.759^&^	0.986		0.851	0.007 (0.000–0.058)
Creatinine (mg/dL)	0.5 ± 0.1 (0.4–0.5)	0.51 (0.2–0.8)	0.4 ± 0.1 (0.4–0.5)	0.45 (0.2–0.7)	0.4 ± 0.1 (0.4–0.5)	0.47 (0.2–0.7)	0.486^$^	0.986		**0.033**	0.016 (0.000–0.085)
AST (U/L)	20 ± 6 (17.1–21.8)	20 (10–32)	24 ± 1 (20.9–29.8)	24 (10–76)	24 ± 11 (20.6–29.1)	24 (7–62)	**0.031** ^&^	0.134	FMF-JİA	0.609	0.067 (0.000–0.172)
ALT (U/L)	19 ± 8 (17.0–23.1)	19 (11–50)	24 ± 10 (21.3–28.8)	24 (9–55)	17 ± 5 (15.7–19.1)	17 (12–33)	**0.001** ^&^	0.013	Control-FMF	**0.001**	0.140 (0.025–0.264)
LDH (U/L)	213 ± 37 (198.6–226.1)	213 (162–318)	229 ± 36 (222.6–249.6)	229 (165–307)	247 ± 45 (231.1–264.9)	247 (140–330)	**0.003** ^$^	0.026	Control-JİA	0.206	0.127 (0.018–0.249)
CK(U/L)	86 ± 33 (71.4–95.9)	86 (20–156)	93 ± 37 (85.6–113.4)	93 (39–182)	85 ± 36 (81.2–108)	85 (55–203)	0.319^&^	0.829		0.258	0.035 (0.000–0.123)
WBC (10^9^/L)	8.3 ± 2.2 (7.2–8.9)	8.37 (3.2–13.8)	7.4 ± 2.7 (6.8–8.9)	7.45 (3.9–17.6)	8.05 ± 2.35 (7267–9295)	8.05 (4.0–16.5)	0.755^&^	0.986		0.865	0.003 (0.000–0.039)
Hb (g/L)	13.5 ± 1.4 (13.1–14.16)	13.5 (11.2–16.7)	13.3 ± 1.1 (12.8–13.6)	13.3 (11.4–16.5)	13.5 ± 1 (13.2–14)	13.5 (12–16)	0.400^$^	0.867		0.194	0.021 (0.000–0.095)
Plt (10^9^/L)	340 ± 84.9 (313.4–376.9)	340 (194–529)	349.5 ± 80.4 (305–365)	349.5 (146–501)	323.5 ± 65.09 (312.1–360.7)	323.5 (247–512)	0.859^$^	0.986		0.734	0.003 (0.000–0.040)
Gelsolin (ng/mL)	25.7 ± 2.8 (24.3–26.4)	25.7 (16.3–29.7)	28.8 ± 2.5 (28–29.9)	28.8 (24.6–33.4)	23.9 ± 2.7 (22.6–24.6)	23.9 (15.8–28.6)	**<0.001** ^&^	**<0.001**	Control-FMF **(***P*** < 0.001**, JIA-FMF (***P* < 0.001**), JIA-Control (p = 0.058)	**<0.001**	0.413 (0.248–0.528)

Data are presented as mean ± standard deviation (SD) and median (minimum-maximum). $ ═ Analysis of Variance (ANOVA); &═ Kruskal–Wallis test. Abbreviations: ALT, alanine aminotransferase; AST, aspartate aminotransferase; BMI, body mass index; BUN, blood urea nitrogen; CI, confidence interval; CK, creatine kinase; FDR, false discovery rate; FMF, familial Mediterranean fever; Hb, hemoglobin; JIA, juvenile idiopathic arthritis; LDH, lactate dehydrogenase; Plt, platelet count; SD, standard deviation; SDS, standard deviation score; WBC, white blood cell count; η^2^, eta-squared.

**Table 2:** Clinical and laboratory characteristics of patient groups

	**JIA (*N* ═ 30)**	**FMF (*N* ═ 30)**	**ki-kare**	***P***
Sex (M/F)	17/13	17/13	1.42	0.49
ESR (mm/h)	25/5	23/7	0.41	0.51
CRP (mg/L)	23/7	22/8	0.08	0.76
ANA (quantitative)	26/3		NA	NA
Fibrinogen (mg/dL)		27/3	NA	NA
Joint involvement		22/8	NA	NA
Uveitis	29/1		NA	NA
MEFV mutation		21/9	NA	NA

Chi-square test was used. Abbreviations: ANA, antinuclear antibody; CRP, C-reactive protein; ESR, erythrocyte sedimentation rate; F, female; FMF, familial Mediterranean fever; JIA, juvenile idiopathic arthritis; M, male; MEFV, Mediterranean fever gene; N, number; NA, not available.

## Materials and methods

This prospective case-control study was conducted between April and September 2025 in the Pediatric and Pediatric Rheumatology outpatient clinics. The study population consisted of children aged 2–18 years diagnosed with JIA or FMF. A total of 60 patients (30 JIA and 30 FMF) were enrolled from the Pediatric Rheumatology Clinic. FMF and JIA cases were identified based on established diagnostic criteria [18, 19] and were classified according to their clinical condition at the time of sampling as JIA exacerbation/remission and FMF attack vs attack-free period. JIA and FMF patients were categorized as active or inactive based on C-reactive protein (CRP) positivity. Additionally, 30 age-matched healthy children aged 2–18 years who attended the Pediatric outpatient clinic for routine follow-up and had no history of chronic disease were included as controls. Exclusion criteria for the control group included prematurity, congenital heart disease, immunodeficiency, metabolic disorders, or any other chronic medical condition. Participants outside the 2–18 year age range were excluded from all groups.

Demographic characteristics, including sex, age, body weight, height, and body mass index (BMI), were obtained from medical records. For patients with JIA and FMF, clinical and disease-related data—including presenting symptoms, sites of involvement, presence of uveitis, *MEFV* gene mutation status, age at diagnosis, duration of follow-up, and medications—were recorded. Laboratory parameters extracted from patient files included erythrocyte sedimentation rate (ESR), CRP, fibrinogen, blood urea nitrogen (BUN), creatinine, aspartate aminotransferase (AST), alanine aminotransferase (ALT), lactate dehydrogenase (LDH), creatine kinase (CK), white blood cell count (WBC), absolute neutrophil count (ANC), absolute lymphocyte count (ALC), hemoglobin (Hb), platelet count, and anti-nuclear antibody (ANA) status.

Serum samples obtained for routine laboratory testing from patients with JIA, FMF, and healthy controls were stored and subsequently analyzed for Gsn concentrations using an enzyme-linked immunosorbent assay (ELISA) according to the manufacturer’s instructions.

### Laboratory measurements

After collection, samples were placed in tubes without anticoagulant and kept at room temperature for 20 min before being centrifuged at 4000 rpm for 10 min at +4^∘^C. The supernatant serum was transferred to Eppendorf tubes and stored at –80 ^∘^C until analysis. Gelsolin concentrations in serum samples were assessed using the Human Gelsolin ELISA Kit (Cat. No. Ck-bio-11601, Shanghai Coon Koon Biotech Co., Shanghai, China), following the manufacturer’s protocol.

Prior to analysis, serum samples were diluted 1:5 according to the manufacturer’s instructions (10 µL serum + 40 µL sample diluent). All standards and samples were assayed in duplicate. The assay sensitivity was 0.1 ng/mL, and the standard curve range was 0–20 ng/mL. Concentrations obtained from the standard curve were multiplied by the dilution factor (×5) to calculate final serum gelsolin levels.

Absorbance was measured at 450 nm using a Rel Assay Diagnostics RL 0505 ELISA reader (Mega Tıp San. ve Tic. Ltd. Şti, Gaziantep, Turkey). A standard curve was generated for each plate and fitted using a four-parameter logistic (4PL) regression model; sample concentrations were interpolated from the curve using ELIASA Software (version 2024.1.25). The intra-assay and inter-assay coefficients of variation were <7% and <10%, respectively (per manufacturer’s data).

### Ethical statement

This study is designed as an Observational Study/Studies to be Conducted with Biochemistry, Microbiology, Pathology, and Radiology Collection Materials such as Blood, Urine, Tissue, and Radiological Images/Studies to be Conducted with Materials Obtained During Routine Examination and Treatment Procedures, in accordance with Erzurum Faculty of Medicine BAEK Decision No. 2025/04-100, and has received approval from the Erzurum City Hospital Ethics Committee. The study was conducted in adherence to the principles of the Declaration of Helsinki. Written informed consent was obtained from the parents or legal guardians of all participants prior to enrollment.

### Statistical analysis

Statistical analyses were conducted using IBM Statistical Package for the Social Sciences (SPSS) Statistics version 20.0. Data are presented as mean ± standard deviation, median (minimum–maximum), count, and percentage, as appropriate. The normality of continuous variables was evaluated using the Shapiro–Wilk test, Kolmogorov–Smirnov test, *Q*–*Q* plots, and assessments of skewness and kurtosis.

Comparisons between two independent groups were made using the independent samples *t*-test for normally distributed variables and the Mann–Whitney *U* test for non-normally distributed variables. For comparisons of continuous variables among more than two independent groups, one-way analysis of variance (ANOVA) was utilized when the normality assumption was satisfied, whereas the Kruskal–Wallis test was employed when it was not. Following ANOVA, post hoc analyses were performed using Tukey’s test when variances were homogeneous and Tamhane’s T2 test when variances were not. For the Kruskal–Wallis test, post hoc comparisons were conducted using the Kruskal–Wallis one-way ANOVA (k samples) procedure. To control for age as a potential confounder, analysis of covariance (ANCOVA) was performed. Effect sizes were reported as eta-squared (η^2^) with 95% confidence intervals.

For the comparison of categorical variables in 2×2 contingency tables, Pearson’s chi-square test was used when expected cell counts exceeded 5, Yates’ continuity-corrected chi-square test was applied when expected counts were between 3 and 5, and Fisher’s exact test was utilized when expected counts were below 3. For categorical comparisons involving tables larger than 2×2, Pearson’s chi-square test was applied when expected counts were above 5, while the Fisher–Freeman–Halton test was used when expected counts were below 5.

To identify independent predictors differentiating JIA from FMF, forward conditional binary logistic regression analysis was performed. Variables with *P <* 0.05 in univariate analyses were considered candidates for the regression model. Results were expressed as odds ratios (OR) with 95% confidence intervals. To address multiple comparisons, the Benjamini–Hochberg false discovery rate (FDR) correction was applied, and FDR-adjusted *P* values were reported alongside unadjusted *P* values.

The discriminative ability of gelsolin for distinguishing between diagnostic groups was assessed using receiver operating characteristic (ROC) curve analysis. The area under the curve (AUC) was calculated along with 95% confidence intervals. The optimal cut-off value was determined using the Youden index, with corresponding sensitivity and specificity values reported.

A two-sided *P* value <0.05 was considered statistically significant.

## Results

A total of 90 children participated in the study: 30 patients with JIA, 30 patients with FMF, and 30 healthy controls. The mean age was 13 ± 4 years in the JIA group, 9 ± 3 years in the FMF group, and 9 ± 3 years in the healthy control group. The overall mean age of the study population was 10.3 ± 4.5 years (range: 3–18 years). Of the participants, 47 (52.2%) were male and 43 (47.8%) were female.

Table 1 presents the associations between Gsn levels and demographic and laboratory parameters in the JIA, FMF, and control groups. Clinical and laboratory characteristics of the patient groups are summarized in Tables 2 and 3.

**Table 3:** Clinical and laboratory characteristics of patients with JIA and FMF

		**JIA**		**FMF**	
	**Mean ± SD**	**Median (min–max)**	**Mean ± SD**	**Median (min–max)**	**P**
ANC (10^9^/L)	4.3 ± 1.9	4.3 (1.4–9.7)	4.3 ± 2.2	3.7 (2.1–12.9)	0.750^ω^
ALC (10^9^/L)	2.9 ± 1.1	2.90 (1.1–5.5)	2.8 ± 0.9	2.74 (0.8–5.0)	0.524^#^
Age at diagnosis (year)	9.4 ± 4,5	10.5 (1–17.5)	6.5 ± 3.3	6.0 (2–14)	**0.010** ^ω^
Follow-up duration (months)	35.0 ± 34.0	24 (4–120)	32.0 ± 29.0	24 (1–132)	0.927^ω^

Data are presented as mean ± SD and median (min-max). The ω symbol denotes the Mann-Whitney *U* test, while the # symbol indicates the independent samples *t*-test. Statistically significant results are highlighted in bold. Abbreviations: ALC, absolute lymphocyte count; ANC, absolute neutrophil count; FMF, familial Mediterranean fever; JIA, juvenile idiopathic arthritis; max, maximum; Med, median; min, minimum; SD, standard deviation.

Twenty-three percent of JIA patients and 26.7% of FMF patients were active at the time of admission. Subgroup analyses comparing active and inactive patients with JIA and FMF, along with correlation analyses between CRP, ESR, ANC, and other parameters, are shown in Tables 4 and 5.

**Table 4:** Clinical and laboratory characteristics of active and inactive JIA patients

		**Active (*n* ═ 7)**		**Remission (*n* ═ 23)**	
	**Mean±SD**	**Median (min–max)**	**Mean±SD**	**Median (min–max)**	***P***
Age (years)	13 ± 6	16 (4–17)	12 ± 4	13 (4–18)	0.431^ω^
Weight SDS	--0.1 ± 2.4	--0.59 (--3.3–4.4)	--2 ± 10.1	--0.2 (--0.4–3.2)	0.750^ω^
Height SDS	--0.1 ± 1.2	0.3 (--1.9–1.0)	0.04 ± 1.4	--0.08 (--1.8–4.2)	0.717^#^
BMI SDS	--0.21 ± 2.02	--0.04 (--3–3.5)	--0.1 ± 1.2	--0.5 (--2.4–2.2)	0.869^#^
BUN (mg/dL)	11 ± 2	12 (9–14)	11 ± 3	12 (7–19)	0.974^#^
Creatinine (mg/dL)	0.5 ± 0.2	0.5 (0.3–0.8)	0.5 ± 0.1	0.5 (0.28–0.8)	0.548^#^
AST (U/L)	19 ± 9	17 (10–32)	20 ± 6	20 (11–31)	0.921^#^
ALT (U/L)	18 ± 6	19 (11–27)	21 ± 9	18 (13–50)	0.676^ω^
LDH (U/L)	228 ± 45	230 (180–318)	208 ± 34	212 (162–266)	0.218^#^
CK (U/L)	70 ± 45	60 (20–156)	88 ± 28	89 (44–153)	0.223^#^
WBC (10^9^/L)	9.5 ± 2.6	9.7 (5.2–13.8)	7.6 ± 1.9	8.2 (3.2–11)	**0.044^#^**
Hb (g/L)	13 ± 1.4	13 (11.2–15.2)	13.8 ± 1.3	13.6 (11.2–16.7)	0.171^#^
Plt (10^9^/L)	418.4 ± 72.7	453 (312–492)	322.9 ± 76.5	328 (194–529)	**0.007^#^**
Gelsolin (ng/mL)	25.4 ± 1.2	25.4 (23.3–27.1)	25.4 ± 3.1	26.05 (16.3–29.7)	0.589^ω^
ANC (10^9^/L)	6.0 ± 2.1	6.4 (3.4–9.7)	3.8 ± 1.6	3.8 (1.4–8.7)	**0.010^#^**
ALC (10^9^/L)	2.6 ± 0.9	3 (1.1–3.8)	3.0 ± 1.1	2.8 (1.3–5.5)	0.372^#^
ESR (mm/h)	34 ± 37	24 (7–112)	5 ± 3	4 (3–12)	0.000^ω^
CRP (mg/L)	21.3 ± 25.3	9 (6–73)	3 ± 0	3 (3–3)	0.000^ω^
Age at diagnosis (years)	9.8 ± 5.5	11 (1.5–16)	9.2 ± 4.3	10 (1–17.5)	0.785^#^
Follow-up duration (months)	35 ± 27	24 (5–72)	35 ± 36	24 (4–120)	0.738^ω^

^ω^*Mann-Whitney U (Z) testi,*^#^ Independent samples *t*-test. Abbreviations: ALC, absolute lymphocyte count; ALT, alanine aminotransferase; ANC, absolute neutrophil count; AST, aspartate aminotransferase; BMI, body mass index; BUN, blood urea nitrogen; CK, creatine kinase; CRP, C-reactive protein; ESR, erythrocyte sedimentation rate; Hb, hemoglobin; JIA, juvenile idiopathic arthritis; LDH, lactate dehydrogenase; max, maximum; min, minimum; n, number; Plt, platelet count; SD, standard deviation; SDS, standard deviation score; WBC, white blood cell count.

**Table 5:** Clinical and laboratory characteristics of active and inactive FMF patients

	**Active (*n* ═ 11)**		**Inactive (*n* ═ 19)**		
	**Mean±SD**	**Median (min–max)**	**Mean±SD**	**Median (min–max)**	***P***
Age (years)	10±5	12 (4–15)	9±3	9 (4–14)	0.265^#^
Weight SDS	--0.6±1.1	--0.5 (--2.5–1)	--0.2±0.9	--0.3 (--2.6–1.2)	0.369^#^
Height SDS	--0.6±0.8	--0.3 (--2–0.3)	--0.3±1.0	0.06 (--2.0–1.8)	0.509^#^
BMI SDS	--0.15±1	0.01 (--1.6–1.3)	--0.1±1.4	--0.1 (--2.8–2.1)	0.962^#^
BUN (mg/dL)	11±2	11 (9–15)	12±3	12 (7–21)	0.584^#^
Creatinine (mg/dL)	0.5±0.17	0.5 (0.2–0.7)	0.4±0.1	0.45 (0.3–0.7)	0.634^#^
AST (U/L)	19±6	18 (11–29)	28±13	25 (10–76)	0.027^ω^
ALT (U/L)	19±5	19 (9–25)	27±10	27 (15–55)	0.033^ω^
LDH (U/L)	236±34	238 (184–284)	236±38	229 (165–307)	0.983^#^
CK (U/L)	78±19	78 (51–110)	107±39	105 (39–182)	0.011^#^
WBC (10^9^/L)	7.9±4.1	6.7 (4.5–17.6)	7.9±2.1	8.2 (3.9–11.4)	0.412^ω^
Hb (g/L)	13.3±1.5	13 (11.7–16.5)	13.3±0.9	13.5 (11.4–14.8)	0.980^#^
Plt (10^9^/L)	301±77.4	277 (212–425)	347.5±79.6	355 (146–501)	0.166^#^
Gelsolin (ng/mL)	28.4±2.2	28.5 (24.6–31)	29.1±2.7	28.9 (24.6–33.4)	0.537^#^
ANC (10^9^/L)	5.0±3.4	3.6 (2.5–12.9)	4.1±1.6	3.9 (2.1–7.9)	0.725^ω^
ALC (10^9^/L)	2.1±0.8	2.1 (1.1–3.4)	3.0±0.9	2.8 (0.8–5)	0.028^#^
ESR (mm/h)	13±7	12 (4–24)	13±17	6 (2–66)	0.103^ω^
CRP (mg/L)	36.6±21.6	33.5 (9–76)	3±0	3 (3–3)	0.003^#^
Age at diagnosis (years)	7.3±4.6	7 (2–14)	6.2±2.8	6 (2.5–12)	0.569^#^
Follow-up duration (months)	40±26	36 (6–84)	29±31	21 (1–132)	0.149^ω^

^ω^*Mann-Whitney U (Z) test,*^#^Independent samples *t*-test. Abbreviations: ALC, absolute lymphocyte count; ALT, alanine aminotransferase; ANC, absolute neutrophil count; AST, aspartate aminotransferase; BMI, body mass index; BUN, blood urea nitrogen; CK, creatine kinase; CRP, C-reactive protein; ESR, erythrocyte sedimentation rate; FMF, familial Mediterranean fever; Hb, hemoglobin; LDH, lactate dehydrogenase; max, maximum; min, minimum; n, number; Plt, platelet count; SD, standard deviation; SDS, standard deviation score; WBC, white blood cell count.

Although CRP and ESR levels tended to be higher in the FMF group compared to the JIA group, these differences did not reach statistical significance (*P ═* 0.51 and *P ═* 0.76, respectively). No significant associations were observed between Gsn levels and ANA positivity, MEFV gene mutations, elevated fibrinogen levels, or the presence of uveitis within the patient groups.

**Figure 1.**
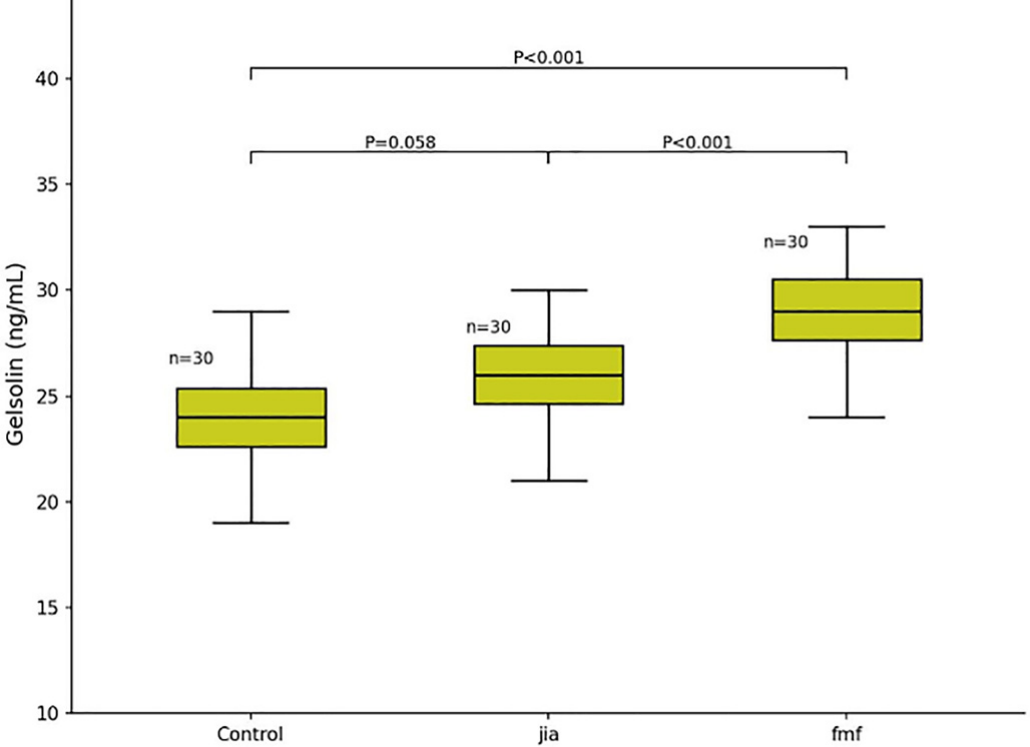
**Serum gelsolin levels in children with juvenile idiopathic arthritis, familial Mediterranean fever, and healthy controls.** Box plots compare serum gelsolin concentrations among healthy controls, patients with JIA, and patients with FMF, with 30 participants in each group. The central line indicates the median, the box represents the interquartile range, and the whiskers denote the data range. Serum gelsolin levels were highest in the FMF group, followed by the JIA group and the control group. The overall between-group difference was statistically significant (*P <* 0.001). Pairwise comparisons showed significantly higher gelsolin levels in the FMF group than in both the control group (*P <* 0.001) and the JIA group (*P <* 0.001), whereas the difference between the JIA and control groups did not reach statistical significance (*P ═* 0.058). Abbreviations: FMF, familial Mediterranean fever; JIA, juvenile idiopathic arthritis.

**Figure 2.**
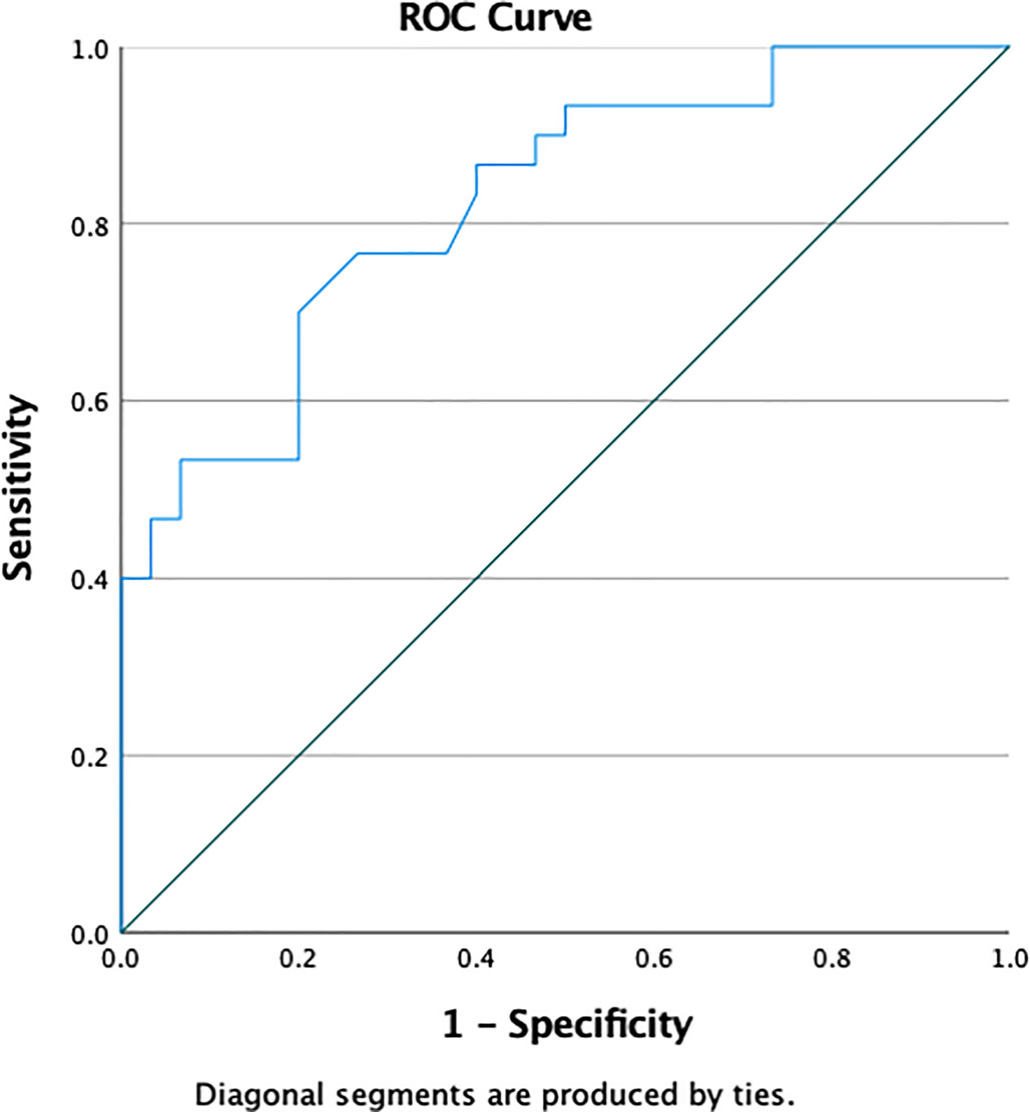
**ROC of serum gelsolin for differentiating FMF from JIA.** The ROC curve illustrates the diagnostic performance of serum gelsolin levels in distinguishing patients with FMF from those with JIA. Serum gelsolin showed good discriminative ability, with an AUC of 0.82 (95% CI: 0.72–0.93; *P <* 0.001). The optimal cut-off value was 27.07 ng/mL, yielding a sensitivity of 0.76 and a specificity of 0.73. The diagonal reference line represents the line of no discrimination. Abbreviations: AUC, area under the curve; CI, confidence interval; FMF, familial Mediterranean fever; JIA, juvenile idiopathic arthritis; ROC, receiver operating characteristic.

Mean Gsn levels were highest in the FMF group (28.8 ± 2.5), followed by the JIA group (25.7 ± 2.8) and the control group (23.9 ± 2.72). The differences among the three groups were statistically significant (*P <* 0.001). Receiver operating characteristic analysis demonstrated good discriminatory ability of Gsn levels, with an AUC of 0.82 (Table 6, Figures 1 and 2).

**Table 6:** ROC analysis of gelsolin levels in JIA and FMF

	**Gelsolin**	**Area under the curve (ROC)**
	**mean** ± **SD**	**Area** ± **SE**	**95% CI for Area (Lower - Upper)**	**cut-off**	**Sensitivity**	**Specificity**	***p***
**FMF**	28.8 ± 2.5	0,8 ± 0,05	0,72 - 0,93	27.07	0.76	0.73	**<0,001**
**JIA**	25.7 ± 2.8						

Abbreviations: AUC, area under the curve; CI, confidence interval; FMF, familial Mediterranean fever; JIA, juvenile idiopathic arthritis; ROC, receiver operating characteristic; SD, standard deviation; SE, standard error.

Colchicine resistance was present in 6.6% of FMF patients. Among these, one patient was receiving interleukin-1 receptor antagonist treatment, and another was on methotrexate (MTX) and corticosteroid therapy. In the JIA group, 56.7% received adalimumab, 46.7% MTX, 26.7% etanercept, 20% corticosteroids, 3.3% leflunomide, 3.3% sulfasalazine, 3.3% azathioprine, 3.3% depo-sillin, and 3.3% folic acid.

When comparing FMF and JIA patients in greater detail regarding age at diagnosis and follow-up duration, the JIA group (10.5 ± 4.5 years) exhibited a significantly higher age at diagnosis compared to the FMF group (6.5 ± 3.3 years) (*P ═* 0.01).

The results of the backward logistic regression analysis, conducted to identify independent risk factors influencing differential diagnosis between JIA and FMF groups, are presented above. In the initial stage of the analysis (Step 1), demographic variables such as age (*P ═* 0.7) and gender (*P ═* 0.5), along with clinical parameters including VA SDS (*P ═* 0.5) and ALT levels (*P ═* 0.1), were not statistically significant and were eliminated from the model. This finding indicates that the diagnostic utility of biochemical parameters is independent of demographic factors. In the final stage of the model (Step 5), gelsolin, AST, and LDH levels were identified as statistically significant and independent predictors of differentiation between FMF and JIA (*P <* 0.05). Specifically, each unit increase in gelsolin levels was associated with a 2.8-fold increase in the probability of a patient being classified in the FMF group (OR: 2.8, 95% CI: 1.5–5.2, *P ═* 0.001). Similarly, increases in AST (OR: 1.1, *P ═* 0.02) and LDH (OR: 1.03, *P ═* 0.01) levels significantly increased the probability of being classified in the FMF group. These findings indicate that high gelsolin levels, in particular, are the strongest independent predictor in the differential diagnosis of the two disease groups (Table 7).

**Table 7:** Backward LR logistic regression analysis (JIA and FMF)

**Analysis step**	**Variable**	**B** ± **S.E.**	**OR**	**95% CI for OR**		*p*
Step 1	Gender	--0.5 ± 0.9	0.6	0.10	3.52	0.57
	Years	--0.04 ± 0.1	0.9	0.73	1.26	0.77
	VA SDS	--0.06 ± 0.09	0.9	0.79	1.12	0.53
	AST	0.09 ± 0.06	1.09	0.97	1.21	0.11
	ALT	0.09 ± 0.07	1.09	0.96	1.25	0.17
	LDH	0.04 ± 0.02	1.04	1.006	1.07	0.02
	Gelsolin	1.1 ± 0.3	3.03	1.60	**5.76**	**0.001**
	Constant	--42.50 ± 13.5	0.00			0.002
Step 5	AST	0.13 ± 0.06	1.1	1.01	1.29	0.02
	LDH	0.04 ± 0.02	1.03	1.00	1.06	0.01
	Gelsolin	1.06 ± 0.3	2.8	1.58	**5.29**	**0.001**
	Constant	--40.09 ± 11.3	0.00			<0.001

Abbreviations: ALT, alanine aminotransferase; AST, aspartate aminotransferase; B, regression coefficient; CI, confidence interval; FMF, familial Mediterranean fever; JIA, juvenile idiopathic arthritis; LDH, lactate dehydrogenase; LR, likelihood ratio; OR, odds ratio; SD, standard deviation; SDS, standard deviation score; SE, standard error; VA, weight-for-age.

## Discussion

In our study, Gsn levels were significantly higher in patients with JIA and FMF compared to the healthy control group, consistent with findings from other studies in the literature [15, 18, 21–24]. In this context, Gsn may serve as a valuable biomarker for evaluating treatment adherence in chronic inflammatory conditions. Gsn is an actin-binding protein involved in cell formation, metabolism, and wound healing processes. Given its known role in arthritis, this study aimed to assess its utility as a biomarker in chronic inflammatory diseases.

This investigation is the first to examine the role of Gsn levels in children with FMF and JIA. Our results indicate that Gsn levels are elevated in children with FMF and JIA compared to the control group. The study by Piktel et al. suggests that impaired gelsolin-actin interactions due to mutations in Gsn can lead to elevated Gsn levels [15].

Consistent with these findings, Gsn levels in our FMF cohort were higher than those observed in other study groups. However, previous studies conducted on adult FMF patients have reported decreased Gsn levels [21, 25]. This discrepancy suggests that age-related differences, disease duration, or inflammatory burden may influence circulating Gsn concentrations. In a study by Feldt et al. involving patients with various forms of arthritis, elevated Gsn levels were found across different arthritis subtypes [26]. Similarly, our study demonstrated that children with JIA exhibited significantly higher Gsn levels compared to the control group, supporting a potential association between Gsn and inflammatory arthritic processes.

Several investigations have explored age-related changes in Gsn levels, indicating that Gsn concentrations may decline with aging, with lower levels associated with frailty, impaired immune function, and reduced tissue repair capacity [12, 15, 20]. These observations underscore the importance of considering age as a potential confounding factor when interpreting Gsn levels and may partially explain discrepancies between pediatric and adult studies.

Prior research has shown that Gsn levels are reduced in animal models of RA, suggesting that the activation of inflammatory pathways contributes to disease pathogenesis and that Gsn may serve as a diagnostic biomarker for conditions characterized by chronic inflammation, such as RA [22]. In contrast to our findings, Osborn et al. reported decreased Gsn levels in adult patients with arthritis, potentially due to the redistribution of Gsn into the inflamed synovial compartment, its binding to actin released from damaged cells, the formation of actin-Gsn complexes, reduced synthesis, proteolytic degradation, or binding to plasma factors [20]. Given its broad anti-inflammatory buffering capacity and actin-binding properties, the reduction of Gsn under chronic inflammatory conditions is hypothesized to contribute to cellular injury and increased mediator release. Indeed, Gsn depletion has been documented in RA [20]. According to the proposed model, the decline in circulating Gsn results from its redistribution to the inflamed synovial space for actin severing. Alternative explanations include decreased production, proteolytic breakdown, or increased binding to plasma proteins [20].

Another study found that Gsn concentrations in juvenile arthritis patients were higher than those in adults with RA or osteoarthritis (OA) and healthy individuals [26]. This observation has been attributed to physiologically higher synovial Gsn concentrations in children and adolescents, as well as potential influences of disease stage, duration, and JIA subtypes on Gsn expression. Supporting this hypothesis, the authors noted that Gsn levels were not evenly distributed across JIA subgroups; persistent oligoarthritis exhibited higher Gsn concentrations, while other subtypes tended toward lower values (below 10 ng/mL) [26]. In line with this, our study also demonstrated elevated Gsn levels in the pediatric cohort compared to healthy controls.

Studies involving FMF patients have shown that, unlike most pathological conditions characterized by decreased Gsn levels, FMF may present with increased serum Gsn. This elevation has been attributed to secondary actin-induced synthesis or release of Gsn from Gsn-actin complexes, similar to observations in rhabdomyolysis [23]. Elevated serum Gsn levels have also been reported in Finnish-type Gsn-related familial amyloidosis (FAF), a condition caused by a single-base mutation disrupting Gsn-actin interactions [24, 27]. Consistent with these findings, our study detected higher Gsn concentrations in children with FMF compared to healthy controls.

In the study by Feldt et al., plasma Gsn levels were inversely correlated with CRP concentrations (r ═ –0.2, *P ═* 0.02), although no significant association was observed with other inflammatory markers [26]. In our study, CRP and ESR values were higher in the FMF group than in the JIA group; however, these differences were not statistically significant (*P ═* 0.5 and 0.7, respectively).

Several limitations of the present study should be acknowledged. First, the prospective case-control design and relatively small sample size limit the ability to draw causal inferences and reduce overall statistical power. Second, as the study population was restricted to pediatric patients with FMF and JIA, the generalizability of these findings to adult populations remains uncertain, constituting an additional limitation.

## Conclusion

In our study, Gsn levels were found to be elevated in children with inflammatory diseases compared to the healthy population. This suggests that Gsn levels in FMF and JIA patients could serve as biomarkers for disease progression; thus, high levels may indicate ongoing inflammation and apoptosis. Long-term, larger-scale studies are needed to further investigate this area.
